# Ayahuasca: pharmacology, safety, and therapeutic effects

**DOI:** 10.1017/S109285292400213X

**Published:** 2024-11-20

**Authors:** Rafael Guimarães dos Santos, Jaime Eduardo Cecilio Hallak

**Affiliations:** 1 Department of Neuroscience and Behavior, Ribeirão Preto Medical School, University of São Paulo, Ribeirão Preto, Brazil; 2 National Institute of Science and Technology Translational Medicine (INCT-TM), Brazil.

**Keywords:** Hallucinogens, ayahuasca, dimethyltryptamine, harmine, mental health

## Abstract

Ayahuasca is a botanical hallucinogen traditionally used for therapeutic and ritual purposes by indigenous groups from Northwestern Amazonian countries such as Brazil, Peru, Colombia, and Ecuador. Ayahuasca is made by the decoction of two plants, which are rich in the 5-HT1A/2A partial agonist dimethyltryptamine or DMT (from the leaves of the *Psychotria viridis* bush) and β-carbolines such as harmine, from the stalks of the *Banisteriopsis caapi* vine. There is an increasing interest in the possible therapeutic effects of ayahuasca, especially for psychiatric disorders (major depression, posttraumatic stress disorder, and substance use disorder). This review summarizes information on the pharmacology, safety, and therapeutic potentials of ayahuasca. Although human experimental and naturalist studies published until now suggest a good safety and tolerability profile, often associated with improvements in depressive and anxious symptoms, there are few controlled studies, with small sample sizes, using only single doses, and with short follow-ups. Potential benefits of ayahuasca should be evaluated in larger samples in both experimental and observational studies and using different doses in controlled trials.

## Historical and cultural notes

**Figure 1.**
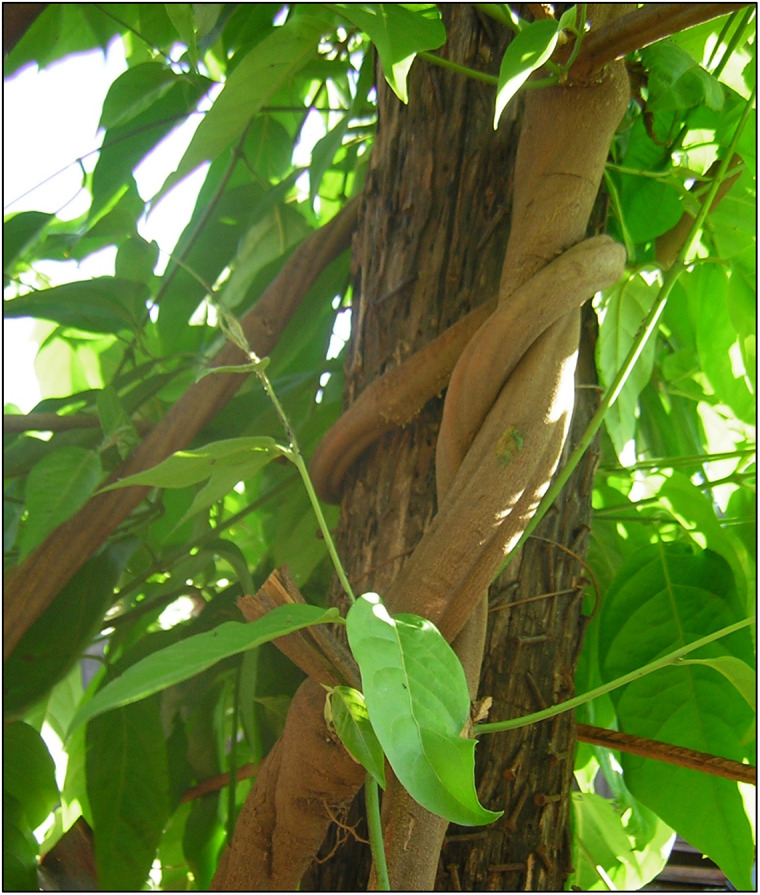
Banisteriopsis caapi.

Ayahuasca is a Quechua term that has the following etymology: *Aya*—means “soul” or “dead spirit”; and *Waska*—“rope” or “vine”. Thus, ayahuasca can be translated as “vine of the souls” or “vine of the dead”. The term refers to *Banisteriopsis caapi* (Figure 1), the vine used as the main ingredient in the elaboration of a psychoactive beverage currently used by more than 70 different indigenous groups of the Amazon pertaining to 20 different language families and spread throughout Brazil, Colombia, Peru, Venezuela, Bolivia, and Ecuador.^1^

In the indigenous context, the beverage is traditionally used to determine the causes and treatments of diseases, consolidate group identity, and promote social order, in rites of passage, and art, divination, and warfare.^2^ ^,^^3^ Depending on the indigenous group, ayahuasca can be used for one or several of these objectives. Importantly, indigenous ayahuasca use needs to be understood within the context of indigenous spirituality and cosmology. Thus, healing, illness, reality, and other concepts are not easily extrapolated to our culture. For instance, according to the indigenous worldview, there is a spiritual aspect to everything that exists and an intimate relationship between humans and nature that can affect health in a broader sense.^3^

Since the beginning of the 20^th^ century, ayahuasca has been used by syncretic religious groups originating in the Amazonian Brazilian states of Acre and Rondônia. The founders of these groups learned to use ayahuasca with the indigenous and mestizo populations of the region and created syncretic groups or churches that have influences from Christianity and Afro-Brazilian and indigenous cosmologies. Among these groups, the most prominent are the *Santo Daime*, *Barquinha*, and *União do Vegetal* (UDV), which use ayahuasca (called *daime*, *vegetal*, or *hoasca*) as a healing tool and for spiritual development. In these rites, members consume ayahuasca usually twice a month. Brazilian legislation allows the ritual use of ayahuasca, and some of these groups (such as *Santo Daime* and UDV) are also present in several European countries, the United States, and Asia.^3^

## The pharmacology of Ayahuasca

### *N,N*-dimethyltryptamine

**Figure 2.**
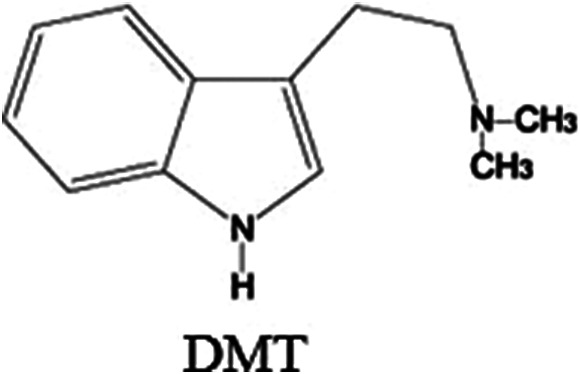
Chemical structure of *N,N*-dimethyltryptamine.

*N,N*-Dimethyltryptamine (or simply DMT; Figure 2) is a tryptamine, like psilocin and psilocybin, and is among the monoamine psychedelics of the indole group, together with lysergic acid diethylamide (LSD), the β-carbolines and ibogaine, being approximately 1000 times less potent than LSD.^4^ DMT was synthesized in 1931 by the Canadian chemist Richard Manske, before it had ever been discovered in any plant. It was first isolated from *jurema* (*Mimosa hostilis*), a plant used ritually by indigenous groups from Northeastern Brazil, by the Brazilian chemist O. Gonçalves de Lima in 1946.^5^ DMT is known to occur in more than 50 plant species,^6^ and it is also present endogenously in animals and humans.^7^

**Figure 3.**
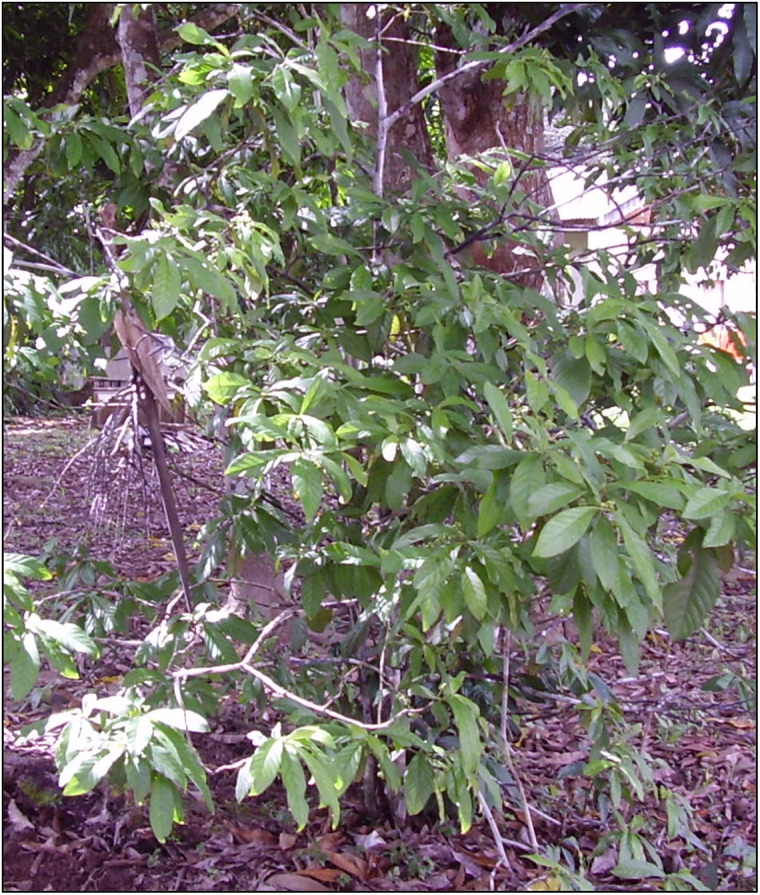
Psychotria viridis.

DMT is also present in the main plants used for ayahuasca preparation: *Psychotria viridis* (Figure 3) in Brazil, Peru, Ecuador, and most places outside South America, and *Diplopterys cabrerana* in Colombia and Ecuador.

DMT has been found to be inactive orally in doses as high as 1 g, but it has been found to be psychoactive after intramuscular administration (0.25-2.00 mg/kg), when inhaled as vaporized free-base (0.2-0.7 mg/kg), and after intravenous administration (0.2-0.4 mg/kg).^8^ ^-^^10^ The intramuscular route produces an experience that initiates around 3-5 minutes and ends after 1 hour.^8^ With intravenous administration or with vaporized/smoked DMT, the subjective effects initiate almost instantaneously (around 30 seconds) and end after 20-30 minutes.^9^ ^,^^10^ Peak concentrations of DMT (100 ng/ml) were reached after 10-15 min following an intramuscular injection of a 0.7 mg/kg dose, and then fell rapidly to baseline levels. After about 45-120 min, DMT levels were undetectable.^11^ By the intravenous route, mean peak value at 2 minutes after a 0.4 mg/kg dose was approximately 90 ng/ml; plasma levels could be measured up to 30 minutes after injection and had virtually disappeared at 60 minutes for all doses (0.05, 0.1, 0.2 and 0.4 mg/kg).^9^

Thus, DMT is rapidly metabolized, and its effects are short-lived. Subjective effects share common features as those of other serotoninergic hallucinogens, including perceptual alterations (mainly visual), increased introspection, improved mood, and less often, anxiety, confusion, and dissociation. DMT and other hallucinogens such as psilocybin and LSD display agonist activity at several serotoninergic (5-HT1A/2A/2C) and nonserotoninergic (dopaminergic and noradrenergic receptors, sigma-1 receptor) receptors, but several preclinical and human studies show that layer V cortical 5-HT2A receptors are mainly responsible for the subjective effects of these drugs.^12^ ^-^^14^

DMT increases serum levels of prolactin, growth hormone (GH), β-endorphin, corticotropin (adrenocorticotropic hormone, ACTH), and cortisol in humans.^9^ ^,^^15^ Other psychedelics such as LSD and psilocybin also increase prolactin, cortisol, and ACTH levels, and these effects seem to be mediated by 5-HT1A/2A receptors.^16^ ^,^^17^

DMT also produces increases in blood pressure, heart rate, and pupillary diameter.^8^ ^,^^9^ ^,^^11^ ^,^^15^ These effects could be mediated by the 5-HT2A receptor, whose activation causes rises in blood pressure and generalized sympathetic activation.^18^ These effects of DMT are also observed for LSD and psilocybin.^16^ ^,^^17^ ^,^^19^

Classical psychedelics such as LSD and psilocybin produce rapid tolerance in humans.^20^ ^-^^22^ Besides tolerance, cross-tolerance also occurs between classic psychedelics.^22^ ^,^^23^ This tolerance is explained by the downregulation and desensitization of 5-HT2A receptors.^24^ ^-^^26^ However, it is difficult to produce tolerance to DMT in animals and humans,^11^ ^,^^27^ ^-^^29^ little or no cross-tolerance occurs between DMT and LSD, and LSD-tolerant individuals show undiminished responses to DMT.^21^ ^,^^29^ The lack of tolerance to DMT could be due to its rapid metabolization^9^ or for affinity in other receptors (while no tolerance was observed for the 5-HT2A receptor, it was observed in the 5-HT2C receptor^4^). In humans, while no tolerance was observed for subjective effects or blood pressure increases after four doses of 0.3 mg/kg intravenous DMT administered at 30 min intervals, increases in ACTH, prolactin, and cortisol levels, and heart rate decreased from the first to the fourth dose.^15^

### Monoamine oxidase inhibition

**Figure 4.**
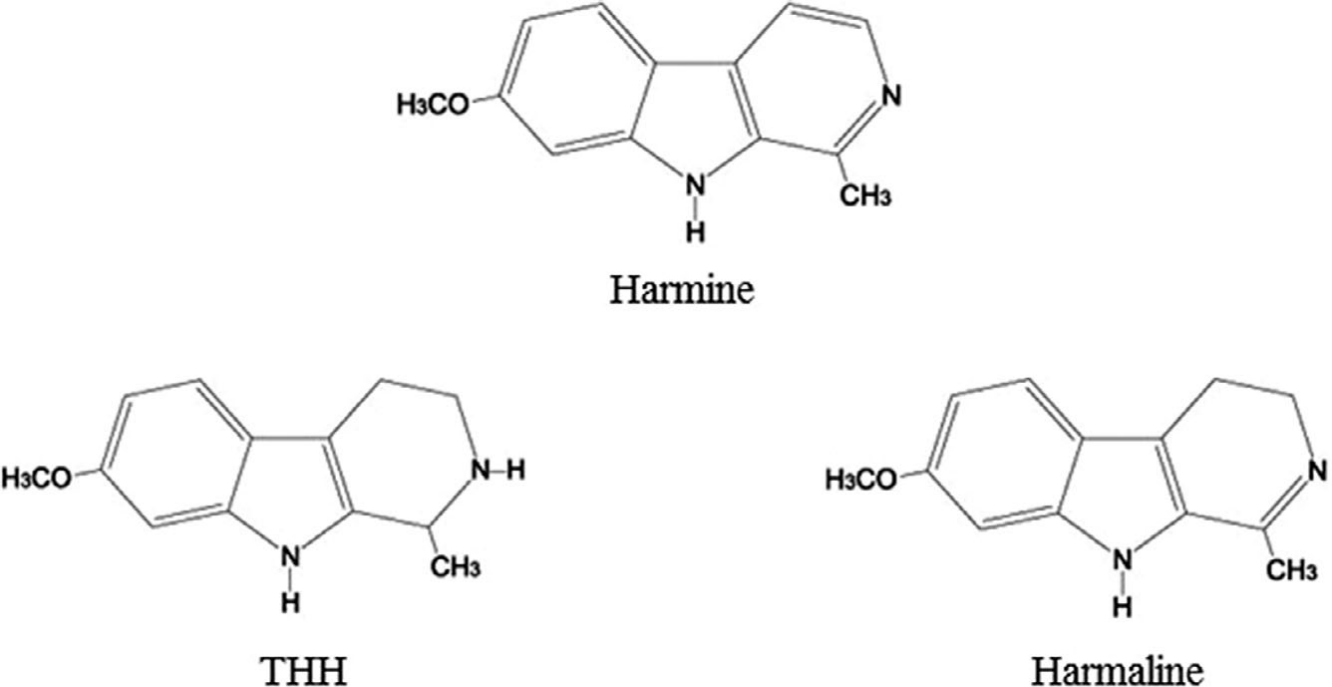
Chemical structures of harmine, THH, and harmaline.

Several liana species of the *Banisteriopsis* genus (Malpighiaceae) are used to produce ayahuasca, which is rich in β-carbolines.^1^ ^,^^2^ ^,^^6^ ^,^^30^ The more commonly used of these species, *B. caapi*, is rich in harmine and tetrahydroharmine (THH), with lower quantities of harmaline and traces of harmol, harmalol, other substances related to the β-carbolines, and other minor compounds (steroids, terpenes, pyronoids).^6^ ^,^^30^ ^-^^32^ The β-carbolines alkaloids are the most abundant compounds in ayahuasca.^6^ ^,^^30^ Harmaline was first isolated from the seeds and roots of Syrian rue (*Peganum harmala*, Zygophyllaceae) by Goegel in 1841, while harmine was first isolated from *P. harmala* seeds by Fritsche in 1847.^6^ The chemical structures of harmine, THH, and harmaline are shown in Figure 4.

Harmine, THH, and harmaline are potent selective, reversible, and competitive inhibitors of the monoamine oxidase (MAO) enzyme, especially of the MAO-A subtype, the form for which norepinephrine, serotonin, and presumably other tryptamines (including DMT) are the main substrates.^30^ ^,^^33^ Ayahuasca inhibited MAO in vitro, which was dose-dependently related to β-carboline content.^30^ Moreover, THH also acts as a selective inhibitor of serotonin reuptake.^34^ Therefore, MAO inhibition and serotonin reuptake by ayahuasca may increase brain levels of serotonin and other monoamines. Harmine and harmaline are metabolized by the enzymes CYP2D6, CYP1A1, and CYP3A4 to hydroxylated harmine and harmaline and their O-demethylated metabolites harmol and harmalol, respectively.^35^ ^-^^38^

Some preclinical studies showed that harmaline and harmine produced hallucinogenic-like behaviors in dogs and cats.^39^ ^,^^40^ In humans, evidence for the β-carbolines to elicit psychedelic has been contested by some authors,^6^ ^,^^41^ while others reported that ayahuasca preparations made only with *Banisteriopsis* species can produce hallucinogenic experiences.^42^ ^,^^43^ Regarding isolated compounds, early studies suggested that intravenous harmine (0.5 mg/kg, 35-45 mg) did not produce psychedelic effects in healthy volunteers,^44^ but higher doses (>150-200 mg) produced psychedelic effects in psychiatric patients (mainly schizophrenic patients), while oral and subcutaneous doses up to 960 mg were inactive.^45^ However, other authors suggested that oral (20-50 mg) and intramuscular (10-20 mg) harmine produced psychedelic effects in healthy volunteers lasting from 3-5 hours (intramuscular) to 6-8 hours (oral),^46^ while others suggested that higher doses of harmine (oral, >8 mg/kg; intravenous, 2 mg/kg) and harmaline (oral, 4 mg/kg; intravenous, 4 mg/kg) are needed to produce psychedelic effects.^47^ ^,^^48^ Oral doses of 120-140 mg harmine would act as sedative, according to some authors.^41^ ^,^^48^ The average (range) harmine and harmaline content (in mg) administered in previous studies involving the administration of ayahuasca to healthy volunteers was 252.3 (204.0–306.0) for harmine and 29.7 (24.0–36.0) for harmaline in one study,^49^ and 95.8 (74.2–114.8) for harmine and 6.5 (5.0–7.8) for harmaline in another study.^36^ Thus, some authors suggested that the total amount of β-carbolines in ayahuasca would not be responsible for the hallucinogenic/psychedelic effects of the brew.^6^ ^,^^41^ The psychoactivity of the β-carbolines (and its possible psychedelics effects) clearly needs to be further investigated.

Regarding safety and adverse effects, harmine and harmaline produced dose-dependent hypothermia in rats,^50^ and bradycardia and hypotension were among the most frequent symptoms reported in human studies.^44^ ^,^^45^ Other effects induced by harmine included nausea, vomiting, tremor, body numbness, and light-headedness,^44^ ^,^^45^ while harmaline induced numbness, physical discomfort, nausea, vomiting, and dizziness.^47^ As with subjective effects, the physiological and adverse effects of harmine and harmaline need to be further elucidated.

In the case of ayahuasca, since pure DMT is not orally psychoactive (doses up to 1 g are inactive in humans^51^) due to peripheral (gastrointestinal and hepatic) metabolization by MAO-A, inhibition of this enzyme by the β-carbolines (especially harmine) allows DMT to reach systemic circulation and the central nervous system.^6^ ^,^^30^ ^,^^36^ ^,^^41^ The threshold dose of harmine necessary to render DMT orally active was established at 1.5 mg/kg (120 mg) by Ott in self-experiments,^41^ who also established that doses of harmaline above 70 mg (1-1.2 mg/kg) could activate tryptamines orally. The β-carbolines/DMT interaction was confirmed in humans by Riba and collaborators,^36^ who showed that ayahuasca administration to healthy volunteers increased urinary excretion of normetanephrine, a metabolite of norepinephrine.

Therefore, considering the limited human data on β-carbolines discussed above and the well-known hallucinogenic effects of DMT, the scientific literature suggests that the main effect of the β-carbolines (mainly harmine) in ayahuasca is to inhibit peripheral MAO-A. However, the human pharmacology of harmine and related β-carbolines is poorly understood, and more research is needed to investigate their effects as isolated compounds and the possible contribution of these compounds to the effects of ayahuasca. For instance, several preclinical studies suggest that harmine may exert antidepressive and neuroprotective effects.^52^ ^,^^53^

### Other ingredients added to the pot

In the indigenous contexts, several species and varieties of the *Banisteriopsis* genus are used to produce ayahuasca, although the more commonly used of these species is *B. caapi.* ^1^ ^,^^2^ ^,^^6^ ^,^^30^ Moreover, depending on the indigenous group or the intention of use, the liana can be used alone, or it can be combined with dozens of other species.^6^ ^,^^42^ For instance, some indigenous groups in Colombia, Peru, or Ecuador may add some species of the Solanaceae family to ayahuasca(*Nicotiana* sp., *Brugmansia* sp., *Brunfelsia* sp.). However, most of these other plants are often used in more restricted contexts, and the most common species used to produce ayahuasca are the DMT-containing plants *P. viridis* (in Brazil, Peru, Ecuador, and most places outside South America) and *D. cabrerana* (in Colombia and Ecuador). Indeed, the legislation in Brazil allows ayahuasca to be produced only with *B. caapi* and *P. viridis*,^54^ and most of the ayahuasca currently being used worldwide is made with these two species.

### Human studies with ayahuasca in healthy volunteers

Research into the pharmacology of ayahuasca in healthy volunteers has been conducted since the late 1990’s. Subjective effects are similar in quality to those of intravenous DMT, but with ayahuasca they are milder and last longer.^10^ ^,^^49^ ^,^^55^ ^,^^56^ Effects include intricate eyes-closed visual imagery, complex thought processes, and a general state of heightened awareness. Overall perceptual, cognitive, and affective processes are significantly modified, in the presence of a clear sensorium. Despite altered perceptions and cognition, users remain aware of their surroundings and can communicate coherently in most of the cases.^49^ ^,^^55^ ^,^^56^

In the first placebo-controlled clinical study assessing the subjective effects and tolerability of three increasing doses of encapsulated freeze-dried ayahuasca (0.5, 0.75, and 1 mg DMT/kg body weight) in six healthy male volunteers with prior experience in the use of the brew, ayahuasca showed a dose-dependent psychological effects which were first noted after 30-60 min, peaked between 60 and 120 min, and were resolved by 240 min.^56^ The time to peak drug concentration (T_max_) for DMT was observed at 1.5 h and coincided with the peak of subjective effects.^36^ The T_max_ for the β-carbolines was similar, with lower plasma concentrations of harmaline. Alkaloid plasma levels returned to baseline levels within 24 hours.^36^ ^,^^49^ Altered physical sensations and nausea were the most frequently reported somatic–dysphoric effects, and volunteers reported some anxiety at peak effects, but the overall experience was regarded as pleasant and satisfactory by five of the six volunteers. One volunteer experienced an intensely dysphoric reaction with transient disorientation and anxiety at the medium dose and voluntarily withdrew from the study. Verbal support was sufficient to handle the situation. After this study, other controlled studies with healthy volunteers involving one single dose^36^ ^,^^57^ ^-^^59^ or two consecutive doses^60^ replicated this pattern of acceptable tolerability, with few cases of challenging psychological experiences.^61^

As with DMT and other psychedelics such as LSD and psilocybin, ayahuasca also produces moderate and transient increases in blood pressure, heart rate, pupillary diameter, and plasma levels of prolactin, GH, and cortisol. These effects are probably mediated by the 5-HT2A receptor.^36^ ^,^^49^ ^,^^57^ ^,^^60^ Moreover, ayahuasca also induced transient modifications in lymphocyte subpopulations, decreasing the percent of CD4 and CD3 cells and increasing natural killer cells. Maximum changes occurred around 2 hours, returning to baseline levels at 24 hours.^57^ ^,^^60^

Electroencephalopgraphic (EEG) studies showed that ayahuasca produced an overall reduction in absolute power in all frequency bands, which was more pronounced in the slow delta and theta bands, while an increase was observed in the relative power of the higher frequency beta bands.^57^ ^,^^60^ ^,^^62^ ^,^^63^ Decreased power density was observed predominantly over the temporo-parieto-occipital junction, temporomedial cortex, and in frontomedial regions, brain areas that are involved in emotional processing and memory processes.^63^ Furthermore, a neuroimaging study using single photon emission tomography (SPECT) showed that ayahuasca increased blood perfusion in the anterior insula/inferior frontal gyrus, anterior cingulate/frontomedial cortex, and in the amygdala/parahippocampal gyrus, brain areas implicated in somatic awareness, subjective feeling states, and emotional arousal.^64^ Studies using functional magnetic resonance imaging (fMRI) showed that ayahuasca increased the activity of the primary visual area during a visual imagery task with a similar magnitude to that observed while seeing a natural image with the eyes open^65^ and decreased the activity of the default mode network.^66^

No tolerance or sensitization seems to occur for subjective and physiological effects after two consecutive doses of ayahuasca 4 hours apart.^60^

## The therapeutic role

As briefly mentioned above, ayahuasca has been used (and still is) for healing and therapeutic purposes in the Amazonian indigenous context for generations and in the last decades also by syncretic churches worldwide.^2^ ^,^^3^ Besides these transcultural traditional uses, from a psychopharmacological perspective, both the brew and its alkaloids have also shown promising therapeutic effects in preclinical and preliminary human studies.

Regarding the β-carbolines, harmine was used in patients with Parkinson’s disease in the 1920s and early 1930s, but interest in this compound disappeared due to the appearance of other drugs.^40^ However, interest in harmine has increased in the last decades. A preclinical study showed that harmine and harmaline could stimulate dopamine release,^67^ and a double-blind, randomized, placebo-controlled trial demonstrated that extracts prepared from the *Banisteriopsis* vine improved motor function in patients with Parkinson’s disease.^43^ Moreover, early studies also suggested that harmaline could be useful in psychotherapy.^47^ Recent preclinical studies have shown that harmine produced antidepressant-like effects in rodents and increased brain-derived neurotrophic factor (BDNF) levels in rat hippocampus.^68^ ^,^^69^ Furthermore, harmine, THH, and harmaline stimulated adult neurogenesis in vitro.^70^ Recent human studies with harmine and the other β-carbolines are lacking.

DMT has shown antidepressant-like effects and enhanced fear extinction in rodents,^71^ which could be promising for the treatment of posttraumatic stress disorder. DMT also has shown neuroplastic effects in preclinical studies.^72^ ^,^^73^ DMT increased neuritogenesis and spinogenesis in vitro and in vivo^72^ and generated new neurons in the mice granular zone.^73^ Further, these mice performed better in memory tests compared to controls, and these effects were blocked by a sigma-1 receptor (S1R) antagonist.^73^ Moreover, DMT has also shown neuroprotective effects mediated by the S1R in a rat model of forebrain ischemia^74^ and in a mice model of Alzheimer’s disease (AD).^75^

In the case of ayahuasca, preclinical studies have shown that the brew has the same profile of effects as those of its isolated alkaloids, with evidence of antidepressant and anxiolytic effects,^76^ ^-^^78^ enhancement of fear extinction,^79^ and anti-inflammatory effects.^78^ Ayahuasca (as well as the beta-carbolines) have also shown promising results in preclinical models of substance use,^80^ ^-^^82^ which is also often observed in naturalist studies with ritual ayahuasca users.^55^ ^,^^82^ ^,^^83^ ^-^^85^

In humans, to the best of our knowledge, there is only a single published trial assessing the possible therapeutic effects of DMT. This was an open-label, phase I study that administered DMT intravenously with psychological support (no psychotherapy was used) to 7 patients with treatment-resistant depression (TRD) and three healthy controls.^86^ The researchers reported a significant decrease of 15% in depressive symptoms one day after the second dosing session (first dose of 0.1 mg/kg DMT followed by a second dose of 0.3 mg/kg two days later). There are other active trials with DMT (intravenous and vaporized) for major depressive disorder and TRD, with promising results that have yet to be published.^87^

In the case of ayahuasca, two trials (one open-label^88^ and one placebo-controlled^89^) have assessed the antidepressant potential of a single ayahuasca dose in patients with TRD, one placebo-controlled trial evaluated the effects of a single ayahuasca dose in a public-speaking test in individuals with social anxiety disorder,^90^ and one single-blind trial assessed the effects of a single dose of ayahuasca in college students with harmful alcohol use.^91^ In all these trials, volunteers were informed about the experimental procedures and ayahuasca general effects before drug sessions, ayahuasca (or placebo) was administered in a comfortable laboratory setting with psychological support (no psychotherapy was used), and follow-ups were performed after the drug sessions. The main information of these trials is shown in Table 1.

**Table 1.** Clinical Trials with Ayahuasca

Reference	Study design/Sample	Main results
Sanches et al. 2016^88^	Open-label 17 patients with TRD 2.2 mL/kg oral ayahuasca (0.8 mg/mL DMT) Psychological support (no psychotherapy)	Significant reductions in HAM-D and MADRS scores at 1-, 7- and 21-days follow-ups
Palhano-Fontes et al. 2019^89^	Double-blind, randomized, parallel-arm, placebo-controlled 29 patients with TRD 1 mL/kg oral ayahuasca (0.36 mg/mL DMT) Psychological support (no psychotherapy)	Significant reductions in MADRS scores at 1-, 2- and 7-days follow-ups and at HAM-D scores at 7-day follow-up
Dos Santos et al. 2021^90^	Double-blind, randomized, parallel-arm, placebo-controlled 17 patients with SAD 2 mL/kg oral ayahuasca (0.68 mg/mL DMT) Psychological support (no psychotherapy)	Significant improvements in self-perception of speech performance (SPSS) during a simulated public-speaking test
Rodrigues et al. 2024^91^	Single-blind 11 college students with harmful alcohol use 1 mL/kg oral ayahuasca (0.61 mg/mL DMT) Psychological support (no psychotherapy)	Significant reductions in days per week of alcohol consumption at 14- and 21-days follow-ups

DMT: Dimethyltryptamine; HAM-D: Hamilton Rating Scale for Depression; MADRS: Montgomery-Asberg Depression Rating Scale; SAD: Social Anxiety Disorder; SPSS: Self-statements During Public Speaking Scale; TRD: treatment-resistant depression.

In the open-label trial with 17 TRD patients, a single ayahuasca dose (2.2 mL/kg; 0.8 mg/mL DMT) induced significant reductions in depressive symptoms from the first hours after ayahuasca intake until 21 days afterwards (61% reduction in the Hamilton Rating Scale for Depression).^88^ Ayahuasca was well tolerated, producing mainly transient nausea and vomiting. These positive results were replicated in a placebo-controlled trial with 29 TRD patients, where a single dose of ayahuasca (1 mL/kg; 0.36 mg/mL DMT), compared to an active, but nonpsychoactive, placebo (designed to simulate the organoleptic properties of ayahuasca: bitter and sour taste with a brownish color), produced significant reductions in depressive symptoms after seven days (57% reduction in the Montgomery-Asberg Depression Rating Scale).^89^ As in the open-label trial, ayahuasca was well tolerated. Moreover, reductions in depressive symptoms were correlated with normalization of salivary cortisol levels,^90^ increases in serum BDNF levels,^91^ and reductions in C-reactive protein plasma levels.^92^

Seventeen patients with SAD participated in a randomized, placebo-controlled, parallel-group trial involving the assessment of their self-perception of performance during a public-speaking test (using the Self-statements During Public Speaking Scale) five hours after ayahuasca intake (2 mL/kg; 0.68 mg/mL DMT) or active, nonpsychoactive, placebo (simulating the organoleptic properties of ayahuasca). Compared with placebo, ayahuasca significantly improved self-perception of speech performance.^93^ Finally, in the single-blind studies involving the administration of a single ayahuasca dose (1 mL/kg; 0.61 mg/mL DMT) to 11 college students with harmful alcohol use, ayahuasca intake was associated with significant reductions in days per week of alcohol consumption at the 14 and 21 days follow-ups (2.90±0.28 vs 2.09±0.41).^94^ However, the quantity of alcohol used by these students was not very high, and the results were no longer significant after Bonferroni correction. Thus, further studies are needed to better investigate the effects of ayahuasca in harmful alcohol use.

In all these trials, no psychotherapy was applied, and ayahuasca was well tolerated, producing mainly nausea, gastrointestinal discomfort, and vomiting. The exact role of psychotherapy in hallucinogen research is still being debated since we still do not know what kind psychotherapy would be better, how many sessions would be necessary before and after drug sessions, who would be allowed to perform it and the effects of placebo and expectancy.^95^ ^-^^97^ Currently, clinical trials (without psychotherapy) exploring the effects of multiple doses of ayahuasca in major depression, comparing the effects of ayahuasca to those of esketamine, and assessing the effects of ayahuasca in other psychiatric disorders (posttraumatic stress disorder, depression in cancer patients) are ongoing in our laboratory.

## Tolerability and safety

### Drug-drug interactions

Drug–drug interactions can be categorized as either pharmacokinetic (when one drug influences the absorption, distribution, metabolism, or elimination of another drug) or pharmacodynamic (modification of the pharmacological effects of one drug by another), and such interactions can be synergistic, additive, or antagonistic. The potential of ayahuasca to interact with other drugs at any of those levels is not well known. By inhibiting MAO-A function, harmine, harmaline, and THH are more prone to produce pharmacokinetic drug-drug interactions, especially concerning the metabolism of serotonin and tyramine. For instance, the concomitant use of ayahuasca with drugs containing high levels of tyramine could produce hypertension since tyramine is a MAO substrate which enhances noradrenaline neurotransmission; and the combination of ayahuasca with other serotoninergic agonists and MAO inhibitors (such as many antidepressants) may result in over-stimulation of the serotoninergic system and may cause a serotonin syndrome, which can be a serious (and even fatal) adverse effect.^98^ ^-^^100^ However, it is important to mention that both effects seem to be rare in naturalist settings and were never reported in experimental/clinical settings.^61^ ^,^^98^ ^-^^102^

Furthermore, harmine and harmaline are metabolized by the enzymes CYP2D6, CYP1A1, and CYP3A4 to hydroxylated harmine and harmaline and their O-demethylated metabolites harmol and harmalol, respectively.^35^ ^-^^38^ Thus, concomitant use of ayahuasca with substances that are metabolized by these enzymes, such as several antidepressants, may also cause pharmacokinetic drug-drug interactions. Concurrent inhibition of serotonin reuptake by antidepressants and THH, together with inhibition of serotonin metabolism by CYP2D6 antidepressants (such as fluoxetine) and MAO inhibitors in ayahuasca, could cause an accumulation of serotonin and a serotonergic syndrome.^103^ Harmine and harmaline have also shown interactions with cholinergic, GABAergic, and glutamatergic neurotransmission in preclinical studies, which are also potential sources of drug-drug interactions.^53^ ^,^^68^ ^,^^69^ ^,^^100^ ^,^^103^

Regarding DMT, as with other psychedelics (LSD, psilocybin), there is the possibility of pharmacodynamic drug-drug interactions with other serotoninergic drugs, especially by competition at the receptor level.^103^ Concomitant administration of DMT and other hallucinogens with serotonin and norepinephrine reuptake inhibitors or MAO inhibitors may reduce the subjective effects of these drugs, probably by increases in serotonin levels and downregulation of 5-HT2A receptors after chronic use, although the evidence is limited and contradictory.^103^ DMT and other hallucinogens are agonists at 5-HT1A/2A/2C receptors, and human studies have shown that 5-HT2A antagonists block most of the subjective and physiological (blood pressure and heart rate, body temperature, neurophysiological effects measured with EEG) effects of these drugs.^12^ ^-^^14^ ^,^^103^ Thus, drugs that are 5-HT2A antagonists will probably reduce the effects of classic hallucinogens, including DMT. A study involving pretreatment with pindolol, a 5-HT1A antagonist, before intravenous DMT administration showed that pindolol significantly increased the subjective effects of DMT,^104^ thus suggesting a “buffering effect” of the 5-HT1A receptor on 5-HT2A-mediated effects. Thus, other drugs that modulate the 5-HT1A receptor could interact with the effects of DMT and other psychedelics.^102^ ^,^^104^ Human studies have also shown that little or no cross-tolerance occurs between DMT and LSD, and that LSD-tolerant individuals show undiminished responses to DMT.^21^ ^,^^29^ ^,^^103^

The possible interactions between DMT and other psychedelics with drugs that act on other neurotransmission systems (dopaminergic, cholinergic, GABAergic, glutamatergic, sigma, endocannabinoid) are not well understood.^103^ Indeed, preclinical studies have shown that DMT acts as a sigma-1 agonist,^73^ ^-^^75^ and ayahuasca acutely increased anandamide plasma levels in patients with SAD.^105^ Further research is needed regarding possible drug-drug interactions with DMT and other hallucinogens.

### General concerns for tolerability and safety

In experimental studies involving the administration of ayahuasca to healthy volunteers^36^ ^,^^55^ ^-^^66^ and patients,^88^ ^-^^91^ ayahuasca was generally well tolerated, and the most common adverse effects reported included nausea, gastrointestinal discomfort and vomiting, transient anxiety, drowsiness, difficulty in concentrating, fear, dissociation/depersonalization and confusion, moderate and transient increases in blood pressure and heart rate, and headaches.^61^ ^,^^100^ Most of these effects were transient and did not need any kind of intervention to be managed, and there were few cases where more intense psychological support was needed. There were no cases where rescue medication and no severe adverse reactions were reported.^61^ ^,^^100^ These results are corroborated by a review of the incidence of adverse events in randomized, placebo-controlled trials with healthy and clinical populations involving ayahuasca administration (n = 108 ayahuasca administrations)^100^ and are similar to results observed in observational/naturalistic studies with ayahuasca users.^102^ ^,^^106^ ^,^^107^ Moreover, observational/naturalistic studies also suggest that long-term ritual use of ayahuasca is not associated with increased psychiatric disorders or cognitive problems.^108^ ^-^^111^ Indeed, some of those studies suggested that long-term use of ayahuasca was associated with less incidence of psychiatric disorders and improved cognition.^108^ ^-^^111^ However, the results of the randomized, placebo-controlled trials should be interpreted with caution since most studies involved small samples, and the observational studies can not establish causal relationships between ayahuasca use and the observed effects. Thus, further longitudinal studies and research with larger samples in clinical populations are needed to better understand the safety and tolerability of ayahuasca.
